# Cytocompatibility Properties of an Herbal Compound Solution Support *In vitro* Wound Healing

**DOI:** 10.3389/fphys.2021.653661

**Published:** 2021-03-26

**Authors:** Peng Zhou, Vanessa Chrepa, Ioannis Karoussis, Michael A. Pikos, Georgios A. Kotsakis

**Affiliations:** ^1^Translational Periodontal Research Laboratory, Department of Periodontics, UT Health San Antonio, San Antonio, TX, United States; ^2^Department of Endodontics, UT Health San Antonio, San Antonio, TX, United States; ^3^Department of Periodontics, National and Kapodistrian University of Athens, Athens, Greece; ^4^Pikos Institute, Trinity, FL, United States; ^5^Department of Periodontics, UT Health San Antonio, San Antonio, TX, United States

**Keywords:** oral rinses, wound healing, chlorhexidine, scratch wound assay, cytotoxicity

## Abstract

The aim of this study was to evaluate the cytocompatibility of an herbal extract compound oral rinse [StellaLife VEGA (SLife)] against relevant human cellular models of oral surgical wound healing. SL was compared to the gold standard for peri-/post-operative oral surgical use, i.e., Chlorhexidine (CHX) and to a commonly utilized essential-oil (EO) based antiseptic rinse. Fibroblasts and primary oral stem cells of the apical papilla (SCAPs) were employed to assess its comparative cytotoxicity to the active comparator antiseptic rinses and its effects on wound healing *in vitro.* In cytotoxicity assays, multiple timepoints were tested ranging from clinically relevant of 60-s rinsing to protracted challenge of up to 5 min, to determine dose-dependent toxicity. The SLife group consistently demonstrated minimal cytotoxicity as compared to active comparators across experimental timepoints and different cells lines. At concentrations up to 20% v/v SLife-challenged fibroblasts and SCAPs demonstrated no significant toxicity as compared to unstimulated controls (*p* > 0.05). When assessing wound healing, a scratch wound assay revealed significantly accelerated cell migration for SLife as compared to CHX (*p* < 0.05). Notably, all active comparator antiseptic rinses affected wound healing responses by significantly reducing total collagen deposition after intermittent “rinsing” intervals that simulated post-surgical oral rinsing. Nonetheless, intermittent as well as continuous challenge of cells with SLife had a positive effect in functional collagen assays. An herbal extract compound-based oral rinse was found to be cytocompatible to cells critical to oral wound healing and to promote fibroblast migration and differentiation, contrary to existing antiseptic rinses that lack selective cytotoxicity.

## Introduction

Herbal anti-inflammatory compounds have recently attracted interest as adjunct treatment in various interventional therapeutic procedures (Simsek et al., 2016; Lee et al., 2017). In comparison to commonly used antimicrobial mouthrinses that are employed in oral surgery for their toxicity against oral bacteria (Bernardi and Teixeira, 2015; Muller et al., 2017), herbal extract-based alternatives primarily act by enhancing wound healing particularly in conjunction with oral surgical treatment (Laugisch et al., 2016). This mode of action provides a different therapeutic focus of post-operative oral therapeutic rinses from bacterial killing to host modulation for uncomplicated wound healing. In part, this alternative approach to pharmacological oral care has been grounded upon a well-established body of literature suggesting that currently used antimicrobial chemotherapeutics, such as chlorhexidine can have negative effects on cellular components of wound healing (Muller et al., 2017). Consequently, a number of plant-based alternatives have been looked at in medicine and in dentistry for their selective antimicrobial properties and ability to effectively switch host response from inflammation to tissue healing (Sharma et al., 2010; Mistry et al., 2014, 2015; Saxena et al., 2015; Younus et al., 2016; Joy Sinha et al., 2017).

Recently published studies provide increasing data that select herbal compounds may have comparable antimicrobial efficacy to established chemotherapeutics (Mistry et al., 2014, 2015; Joy Sinha et al., 2017). Specifically, the extracts of *Azadirachta indica* (Neem) have been shown to have comparable antimicrobial activity to 2% chlorhexidine or sodium hypochlorite against *Enterococcus faecalis, Streptococcus mutans*, and *Staphylococcus aureus.* Further validation in humans showed that calendula delivered in solution for mouthwashes was effective in reducing dental plaque and gingivitis adjunctive to scaling, while *Echinacea* extract had antimicrobial efficacy against respiratory bacteria (Sharma et al., 2010; Khairnar et al., 2013). Nonetheless, antimicrobial activity is only one of the two properties that an oral compound needs to possess for oral wound healing. Selective cytotoxicity is equally as important in order to selectively target toxicity to bacteria, while minimizing adverse toxic effects to host reparative cells (Kotsakis et al., 2016; Muller et al., 2017). This latter aspect has not been prioritized in the past because of a larger focus on antimicrobial effects of existing chemotherapeutics for oral wound healing. For example, the most commonly utilized post-surgical rinse of chlorhexidine is highly cytotoxic to fibroblasts and osteoblasts at concentrations as low as 0.12%. However, because of the ubiquitous presence of bacteria in the oral environment, which may complicate wound healing if they colonize the wound margins, it is clinically often employed based on antibacterial effect potency. Generally, a trade-off exists with current chemotherapeutics having increasing cytotoxicity, while their antibacterial efficacy increases. In response, the study of herbal compounds with wound healing properties has gained interest within medical community to possibly beat that trade-off. In fact, *Echinacea* extracts (Sharma et al., 2010) have demonstrated antibacterial effects at non-cytotoxic concentrations of extract, which appear to be due to multiple components rather than the individual chemical compounds.

The concept of herbal compounds to accelerate wound healing has recently been introduced in dentistry for surgical recovery (Tatch, 2019) and a new homeopathic oral rinse containing herbal extracts of *A. indica*, *Echinacea*, *Calendula* and propolis [StellaLife VEGA^®^ Oral rinse (SLife), Northbrook, IL, United States^∗^] has shown promising potential for facilitating wound healing in the oral mucosa (Fujioka-Kobayashi et al., 2020). The only available study assessing the cytocompatibility of this herbal extract compound oral rinse (SLife) reported that it demonstrated less toxicity than 0.12% chlorhexidine, which is the most commonly used antiseptic for post-surgical oral care (Fujioka-Kobayashi et al., 2020). Further, gene expression analysis was suggestive of possibly accelerated wound healing as compared to control by upregulation of collagen type I transcription (Fujioka-Kobayashi et al., 2020). In comparison, our study extended to the investigation of cytotoxicity to multiple different cellular populations including primary oral stem cells, which are more sensitive probes of toxic biomaterials. Further, the present study included functional wound healing assays, i.e., the scratch wound assay and a functional collagen deposition assay, which provide insights into the activity of the mouthwash beyond viability. Collectively, these results are appealing and contribute to the hypothesis that due to their chemical-free formulation, these compounds may lack the adverse effects common to existing therapeutic oral rinses and that they in fact support wound healing. Therefore, there is heightened interest in the clinical assessment of these herbal compounds. Nonetheless, except for the aforementioned study little is known about the cellular effects of SL and how it affects functional wound healing. Because oral surgical wounds take several days to completely close, cytocompatibility of rinses prescribed post-operatively is important and its absence may impair wound healing and lead to complications.

Thus, the primary objective of this study was to evaluate the cytocompatibility of this herbal extract compound against relevant human cellular models of oral surgical wound healing. The hypothesis to be tested is that the SLife Oral Rinse will perform superior to the clinical gold standard of 0.12% Chlorhexidine.

^∗^The uses for this product are based on traditional homeopathic practice. These statements have not been reviewed by the Food and Drug Administration. All StellaLife products are FDA registered, cGMP and HPUS compliant.

## Materials and Methods

Experiments were performed using L929 and NIH/3T3 murine fibroblasts, and oral stem cells of the apical papilla (SCAP). Briefly, following institutional review board approval, the cells were isolated from a tooth extracted from a 18-year old male dental patient. The tooth was maintained in our IRB-approved biorepository, which does not maintain identifiers and has been approved for a waiver of written consent for collection of patient biological specimens, research and clinical data without identifiers. SCAPs were characterized using flow cytometry for stem cell marker (MSC) expression, and multi-lineage differentiation experiments using osteogenic and adipogenic differentiation media as previously described (Chrepa et al., 2017). SCAPs were validated by assessing the co-expression of the MSC markers CD73, CD90, and CD105 while being negative for the endothelial marker CD45, as previously described (Chrepa et al., 2015, 2017). Fibroblasts were incubated in Dulbecco’s modified Eagle’s medium supplemented with 10% fetal bovine serum and 1% penicillin/streptomycin (DMEM+) and SCAP cells were grown in Minimum Essential Medium α supplemented with 10% fetal bovine serum and 1% penicillin/streptomycin (α-MEM+). All cells were grown at 37°C in a humidified atmosphere of 5% CO_2_. Cells were routinely passaged at 80% confluency and were used between passages P3 and P6 for fibroblast experiments and P3 and P4 for SCAP experiments. Two commonly used commercially available oral rinses were used as controls; an essential oils-based solution (EO; Listerine, Johnson & Johnson, Lititz, PA, United States) and 0.12% Chlorhexidine digluconate (CHX; prescription oral rinse compound) and compared against the test StellaLife Vega rinse (SLife; StellaLife VEGA^®^ Oral rinse, Northbrook, IL, United States).

### Cytocompatibility Assessments

Little is known about the cytocompatibility of SLife, but the only available study has shown that cell viability of gingival fibroblasts was minimal after 5 min of stimulation with 100% SLife solution (Fujioka-Kobayashi et al., 2020). To determine the maximum non-toxic (MNTD) dose of the solution we employed a concentration curve of SLife ranging from 0% (100% DMEM+) up to 60% (60% SL/40% DMEM+) v/v to challenge L929 fibroblasts for 5 min. Briefly, cells were grown on lysin-coated coverslips in 24-well plates at a density of 10^5^ cells/well and challenged for 5 min with either the SLife at different concentrations, CHX and EO active comparators at 10% v/v concentrations, or 10% v/v DMSO as positive cytotoxicity control. Cells grown in DMEM+ served as negative controls. Immediately after the 5-min challenge, cells were then washed twice in PBS and stained with a Zombie Red fixable viability dye (Zombie Red^TM^, Biolegend, San Diego, CA, United States) for 15 min at room temperature in the dark at manufacturer recommended dilution. This amine reactive fluorescent dye is only permeant to cells with compromised membranes, thus providing specific staining of dead cells. Following staining, cells were then fixed in 10% neutral buffered formalin and washed in PBS before blot drying and mounting on glass slides for imaging. Fluorescent images were captured at 624 nm using a 20X objective using a BZ-x800 Keyence microscope.

Subsequently, we assessed cellular viability after clinically relevant oral rinse application time (60 s) using primary stem cells (SCAPs) that are more susceptible to cytotoxic challenge as a more sensitive cytotoxicity model. To avoid quenching of cellular viability dyes by the colored rinses, SCAPs were grown in 6-wells until 80% confluency and loaded with fluorescently conjugated nanocrystals. These nanocrystals were conjugated to a custom targeting peptide that delivers green-fluorescence (525 nm), can be traced through several generations, and are not transferred to adjacent cells in a population (Qtracker^TM^ 525 Cell Labeling Kit, Invitrogen, Carlsbad, CA, United States), thus eliminating any quenching effects of the rinses with the dyes in colorimetric assays. Following incubation with the 10 nM nanocrystals for 5 h, cells were washed with warmed α-MEM+ three times. Cells were then trypsinized and seeded on lysin-coated coverslips in 24-wells as described above. After 24 h incubation, media were aspirated and the cells were challenged with 100% v/v SLife, CHX, EO, or PBS (control) for 60 s to stimulate oral rinsing. Subsequently, all coverslips were washed with PBS x3 times and then fixed in 10% neutral buffered formalin for 15 min at room temperature and mounted for fluorescent imaging as described above.

### Wound Healing Assays

To mimic cell migration during wound healing we employed a well-established scratch wound assay (Liang et al., 2007). L929 fibroblasts were seeded in 6-well plates as described above until they formed confluent monolayers. To examine cell migration rates during wound healing, scratch wounds were created by scraping a 800 μm wide scratch in the center of the plate using a P200 pipette tip and the excess cell material removed by wash with PBS to remove any non-adherent cells (Liang et al., 2007). Media was switched to DMEM without FBS to prevent cell proliferation, and a permissible non-toxic dose of 10% v/v SLife as determined above was continuously added to the test wells diluted in DMEM to assess whether SLife alters wound closure. A 10% v/v CHX group was used as a negative control and DMEM media served as positive controls. Cells were visualized using phase-contrast microscopy at baseline and incubated at 37°C for 48 h to assess migration toward the wound. At various points throughout the incubation with the various incubation conditions (0 h, 2 h, 24 h, and 48 h), the width of the cell free zone was measured at five predetermined, representative regions of interest along the wound. Experiments were repeated in triplicate and the mean and standard deviation for all wound width and area measurements within conditions was calculated. Migration data was analyzed as wound width data over time to calculate the rate of wound closure and the percent migration of cells at various timepoints to provide an even more clear picture of the differences in cell migration and repair. Percent migration was calculated as follows: Pm = {[(wound width *T* = 0)−(wound width at measured timepoint)]/(wound width *T* = 0)} × 100.

To functionally assess collagen matrix deposition from *in vitro* cultured fibroblasts challenged with SLife we assessed total collagen deposition by L929 fibroblasts using a Sirius red assay (Khan et al., 2020). L929 cells were seeded in 6-well plates and incubated in DMEM+ until confluent. Subsequently, media was changed every 3 days for 14 days total and during every media change the cells were washed with 100% v/v SLife, CHX or EO for 60 s to simulate oral “intermittent oral rinsing.” Following each 60 s rinse the cells were washed once in pre-warmed PBS, DMEM+ was added and incubation continued at 37°C in a humidified atmosphere of 5% CO_2_. DMEM+ washes were used as positive controls. Fibroblasts seeded with DMEM+ plus a 10% permissive dose of SLife for the entire experimental period were used as a “continuous” test group. After 14 days of incubation, cells were fixed in 100% methanol for 10 min and subsequently stained for total fibrillar collagen using a Sirius red dye according to manufacturer recommendations (Sirius Red/Fast Green Collagen Staining Kit, Chondrex Inc., Woodinville, WA, United States). Briefly, 300 μl of dye was added in each well and incubated at room temperature for 30 min. The stained cells were washed with distilled water five times until water ran clear and left to dry. Subsequently, cells were imaged at an inverted microscope using a 20x objective. Following imaging 500 μl of extraction buffer were added on each sample and gently mixed by pipetting until the color was eluted from the sample. The eluted dye solution was then transferred to 96-well plates, and the absorbance values were captured at 540 nm and 605 nm with a spectrophotometer. Subsequently, the amount of collagen was calculated by the color equivalence standards for collagen.

### Analysis

Summary statistics were calculated using means and standard deviations. For intergroup comparisons to examine the strength of relationship between different conditions two-way repeated measures ANOVA was implemented in Graph Pad Prism 8.4.1. Data of the test and control conditions were compared to examine the amount of variance existing between these wound healing conditions and when ANOVA results were significant, pairwise comparisons were implemented with *post hoc* tests using Bonferroni corrections for adjusted alpha levels. When multiple timepoints were assessed, repeated measures ANOVA models were implemented for each condition and timepoints followed by appropriate *post hoc* tests as indicated.

## Results

### Cytocompatibility Assessments

**FIGURE 1:**
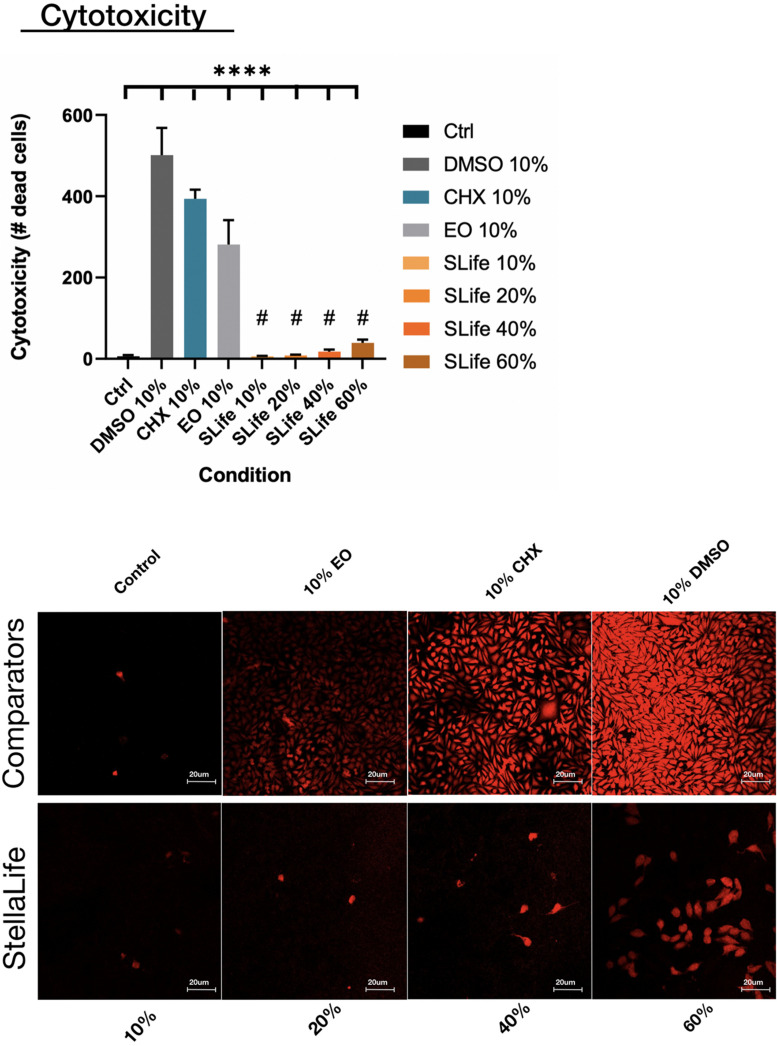
The number of dead cells taking up Zombie red dye were counted after 5 min of incubation with either of the oral rinses. Fluorescent images demonstrated limited toxicity of SLife even up to 40% v/v concentrations as compared to the pronounced cytotoxicity of active comparators at 10% v/v dilutions. ****Designates highly significant difference (*p* < 0.001) to control. ^#^Designates statistically significant difference (*p* < 0.05) to active group comparators.

**FIGURE 2:**
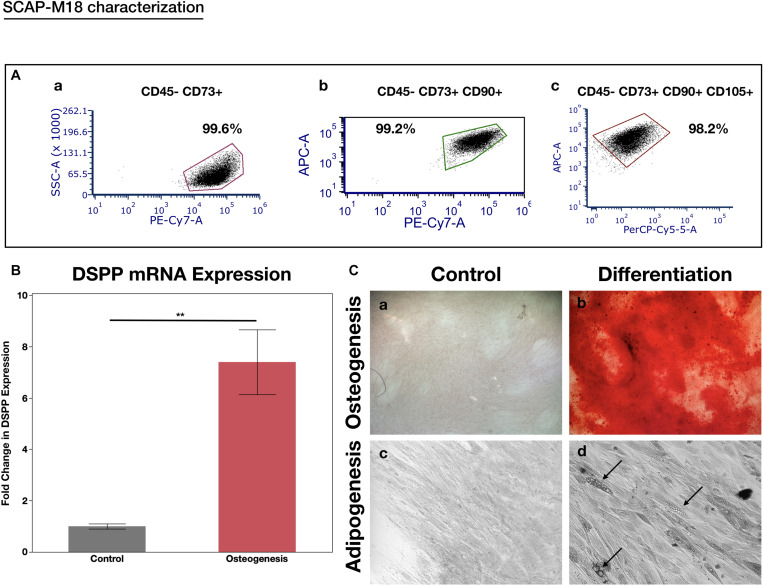
SCAP cell line characterization. **(A)** Flow cytometry expression of the MSC markers in the CD45- population: (a) CD73, (b) CD73 co-expression with CD90, and (c) co-expression of CD73, CD90, and CD105. More than 98% of the cells formed a cell niche and expressed all three stem cell markers while being negative for the endothelial marker CD45-, indicating their stemness. **(B)** RT-PCR analysis of DSPP gene after inducing the cells with osteogenesis media for 14 days. There was a significant 7-fold increase in DSPP expression after osteogenic induction. **p < 0.05. **(C)** Osteogenic (a,b) and Adipogenic (c,d) induction of SCAPs with light microscopy 10x. Alizaring red staining used to depict the mineralization nodules (a,b). Black arrows point to lipid droplets (d).

**FIGURE 3:**
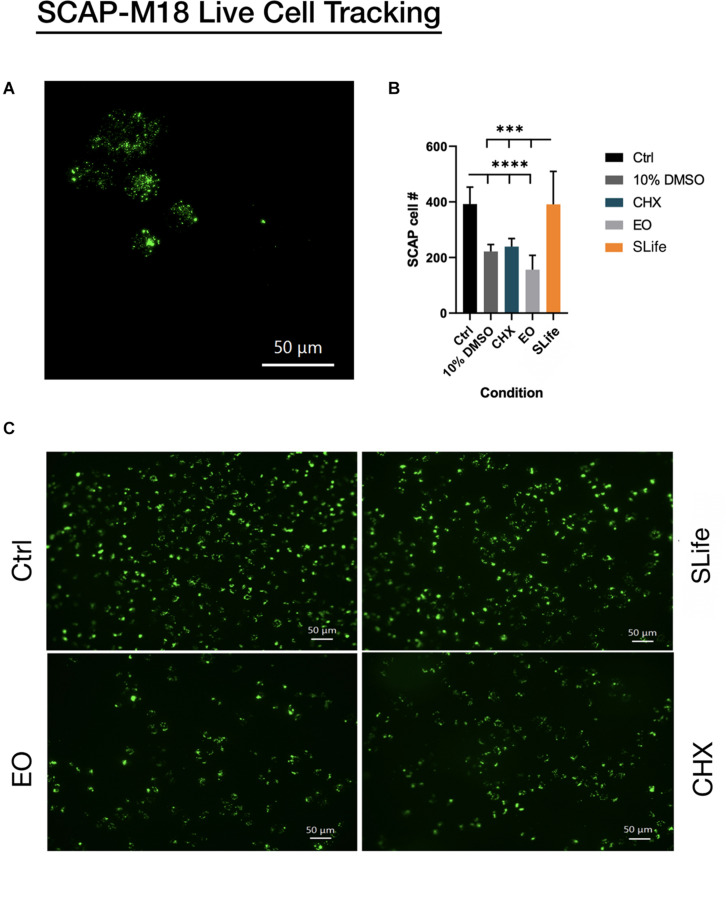
Fluorescent nanocrystals were used to label stem cells of the apical papilla for live cell tracking. **(A)** SCAP cells loaded with nanocrystals can be seen at 40x magnification under fluorescence prior to challenge. **(B,C)** SCAP cells were stimulated with each of the oral solutions: PBS; SLife; EO and Chlorhexidine for 1 min, and then imaged by fluorescent microscopy (20x). **(B)** Lower asterisks in the bar chart represent significant differences between the control condition and selected conditions. Upper asterisks represent significant differences between the SLife group and selected conditions.

When assessing the concentration dependent cytocompatibility of SLife, it was found that concentrations of up to 20% v/v of SLife did not demonstrate increased cytotoxicity as compared to culture media unstimulated controls for up to 5 min of incubation (*post hoc p* > 0.05). Even at concentrations of 40–60% v/v the cytotoxicity of SLife was significantly less than what noted for 10% v/v solutions of CHX or EO in fibroblast cultures (Figure 1). Subsequently, to increase translational potential, we challenged a primary oral stem cell population for 30 s to simulate oral rinsing regimens. Following stem cell isolation as described in the methods, the SCAPs were stained with antibodies against known mesenchymal stem cell markers prior to experiments to assess the purity of the population as previously described (Chrepa et al., 2017). Briefly, following multi-color flow cytometric analysis using a BD FACSAria cell sorter instrument, more than 98% of cells co-expressed the MSC markers CD73, CD90, and CD105 while being negative for the endothelial marker CD45 (Figure 2). The SCAPs also demonstrated robust adipogenic and osteogenic differentiation (Figure 2) after staining with oil red-O and alizarin red, respectively. Subsequently, SCAPs cells were labeled with fluorescent nanocrystals for live cell tracking as shown in Figure 3. Compared to PBS control, SLife treated cells showed similar cell density, indicating that a clinically relevant 30-s challenge of 100% SLife has no obvious cytotoxicity to SCAP cells (*p* > 0.05). As expected, the stimulation of EO and CHX killed cells and showed pronounced toxicity to SCAP cells (Figure 3) (*p* < 0.001). This result demonstrated that SLife has superior cytocompatibility as compared to CHX and EO when assessing oral stem cell responses *in vitro*.

### Wound Healing

**FIGURE 4:**
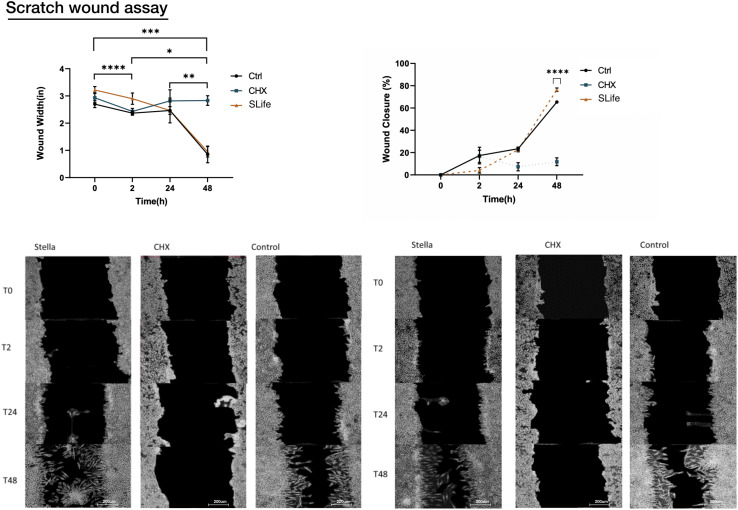
Images of the scratch wounds at two representative regions of interest for each of three conditions are shown at various timepoints following initial incubation (0 h, 2 h, 24 h, and 48 h). SLife group was compared to active comparator CHX as a positive cytotoxic control and against a media only control condition to monitor wound healing in the absence of external factors. Because of cytotoxic effect the CHX condition indicated a wound that remains relatively the same size throughout the assay with exfoliation of cells noted at 2–24 h, which hindered cell migration. SLife led to a slightly more rapid wound closure compared the control, although not significantly different. Asterisks designate significant differences *p* < 0.05. Ascending numbers of asterisks used to differentiate comparisons between increasing timepoint intervals.

Using an established scratch wound healing model, the migration of cells toward the leading edge of the scratch differed largely between SLife and the active comparator. When adding chlorhexidine, cells at the wound edge demonstrated cell death by 24 h and led to increase of the wound margin by 48 h post-intervention (*p* < 0.01). Conversely, wound closure in the SLife progressed exponentially (Figure 4) and at a slightly increased rate as compared to media positive controls although not significantly different. Figure 4 presents a line graph comparing the decrease in wound area over time where it is evident that wound area in the SLife and media-only control conditions decreases rapidly after 24 and 48 h, while the CHX condition shows a substantial inhibitory effect against wound healing at these timepoints. Percent migration for each timepoint suggests that there seems to be significant migration to close the wound in both the Control and SLife conditions (all *p* < 0.01) and that after the 24-h timepoint migration to closure accelerates much more rapidly. SLife shows a slightly increased migration rate compared to the Control, which was not significant. This data suggests that compared to normal conditions, SLife provided similar or slightly faster wound healing and repair but the difference seems to be minor. While SLife supported wound healing in this *in vitro* model, CHX greatly inhibited as evidenced by a constant wound width and thus a lack of cellular migration to close the wound.

**FIGURE 5:**
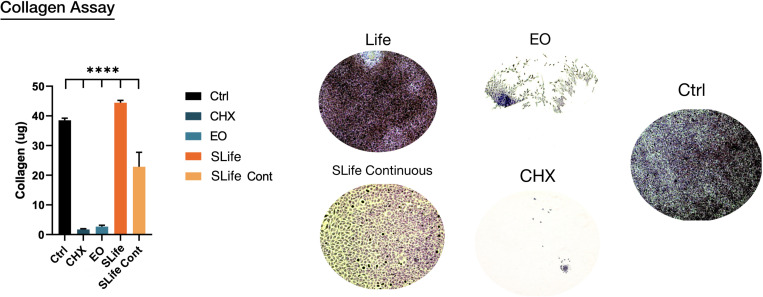
Representative samples showing total collagen (ruby red stain color) deposition in each group. Areas of cell exfoliation appear as white background. ****Designates highly significant difference (*p* < 0.001) to control.

To complement the scratch wound healing model, another functional assay was performed to assess if oral rinsing affects collagen deposition, which is a critical part of wound healing. Intermittent “rinsing” of the cells every 3 days was used to simulate clinical conditions and results showed that SLife significantly increased total collagen deposition by nearly 5 μg more as compared to supplemented media positive controls. In contrast, EO and CHX exhibited cytotoxic effects that led to cell death and vastly reduced collagen deposition. Interestingly, the continuous presence of a permissive 10% v/v concentration of SLife in the culture led to at least 8-fold increased collagen content as compared to the active comparator groups (*post hoc p* < 0.001) (Figure 5).

## Discussion

The current standard of care for the prevention of post-surgical wound healing complications is the use of antiseptic oral rinses (Powell et al., 2005). Current therapeutic oral rinses that are typically prescribed after oral surgical procedures are primarily aimed at antimicrobial effects and not wound healing. In reality, a trade-off exists when antiseptic rinses are employed for oral surgical wounds; with high antimicrobial effects the toxicity to host reparative cells also increases. For instance, because of its known cytotoxicity, chlorhexidine is often empirically prescribed to start at 24 or 48 h post-surgery to avoid incision line healing impairments (Faria et al., 2007; Giannelli et al., 2008; Kotsakis et al., 2016). There is a trend in medical practice to switch to herbal compounds to facilitate uncomplicated wound healing with selective cytotoxicity. The herbal compound rinse investigated in this study has four key components that are independently well established for their antibacterial efficacy and pharmacological effects that support cutaneous wound healing: *A. indica* (Neem), *Calendula*, *Echinacea*, and *Plantago* (Sharma et al., 2010; Khairnar et al., 2013; Mistry et al., 2014; Pazyar et al., 2014; Joy Sinha et al., 2017). Nonetheless, little is known about potential synergistic effects within the compound. Currently, there is only one cellular investigation of SLife, which suggested that it had superior cytocompatibility as compared to Chlorhexidine *in vitro* (Fujioka-Kobayashi et al., 2020).

Findings of the present study are supportive of the results of the aforementioned study showing that in clinically relevant exposure times SLife has no toxicity to either fibroblasts or oral stem cells. Further, SLife demonstrated a slight augmentation of wound healing in functional assays, however, this difference to unstimulated controls was not statistically significant. Nonetheless, these findings were diametrical to cellular responses to existing antiseptic rinses, which were invariably cytotoxic even in small concentrations. *In vitro* cytotoxicity experiments demonstrated that even at concentrations as low as 10% v/v CHX was almost as cytotoxic as DMSO, a well established toxicity control substance, after 5 min of exposure time. Further, CHX fully inhibited wound closure in a scratch wound assay model due to direct toxicity as evidenced by exfoliation of cells at the wound margin after 24 h. This is consistent with the clinical observations of delayed wound healing with use of CHX (Faria et al., 2007; Bernardi and Teixeira, 2015; Muller et al., 2017). Results of uninterrupted wound healing in the presence of SLife support a paradigm shift for oral wound healing. With comparable antibacterial efficacy to antiseptic oral rinses (Khairnar et al., 2013; Mistry et al., 2015; Saxena et al., 2015; Joy Sinha et al., 2017), herbal extracts also demonstrate selective cytotoxicity by supporting wound healing as evidenced by our results.

Nonetheless, because limited information is available on the comparative antimicrobial efficacy of the herbal compound as compared to CHX and EO, antimicrobial studies using clinically relevant biofilms are necessary for a comprehensive comparison of clinical efficacy. Ultimately, this selective cytotoxicity could facilitate uncomplicated wound healing and reduce adverse events. In fact, a SLife oral kit that includes the herbal compound extract oral rinse as well as an additional herbal extract compound with analgesic activity delivered as a sublingual spray and gel has demonstrated human efficacy for inflammatory pain management in a prospective study of thirty-four surgical dental patients (Lee and Suzuki, 2019). The authors found that use of the SLife reduced opioid consumption by 1.5 tablets per day on average (Lee and Suzuki, 2019). Thus, this work supports that herbal anti-inflammatory compounds may have a significant clinical effect for the management of post-operative inflammation after oral surgical procedures by supporting cellular responses to surgical trauma and enabling uninterrupted wound healing.

## Conclusion

Results of the present study show that an herbal extract compound-based oral rinse did not demonstrate toxicity to fibroblasts and oral stem cells when applied for clinically relevant exposure times. Contrary to currently used oral antiseptic rinses that impaired wound healing as an adverse event, it supported wound healing comparable to control tissue culture conditions. Thus, the potential to improve oral wound healing warrants further investigation in *ex vivo* models including oral bacteria and in well controlled human studies to assess clinical effects.

## Data Availability Statement

The raw data supporting the conclusions of this article will be made available by the authors, without undue reservation.

## Ethics Statement

We used an established protocol for collection of extracted teeth in our biorepository and cell isolation that was approved by the Ethics Committee of UT Health San Antonio.

## Author Contributions

GK designed the study and drafted the manuscript. PZ, GK, and VC performed the experiments and collected the data. GK and IK analyzed and interpreted the data. GK, IK, and MP contributed to the discussion of the manuscript. All authors revised the manuscript, accepted the final draft, and share responsibility for the data presented.

## Conflict of Interest

The authors declare that this study was partially supported by a research grant from StellaLife Inc. (Northbrook, IL, United States) who is the manufacturer of the StellaLife Vega Oral Rinse. The funder was not involved in the study design, collection, analysis, interpretation of data, the writing of this article or the decision to submit it for publication.
